# Atrial Fibrillation Catheter Ablation: Overcoming Complications and Improving Success

**DOI:** 10.19102/icrm.2017.081004

**Published:** 2017-10-15

**Authors:** Sunil T. Mathew, Sunny S. Po

**Affiliations:** ^1^Cardiovascular Section, Department of Veterans Affairs Medical Center, Oklahoma City, OK; ^2^Heart Rhythm Institute, University of Oklahoma Health Sciences Center, Oklahoma City, OK; ^3^Cardiovascular Section, Department of Medicine, University of Oklahoma Health Sciences Center, Oklahoma City, OK

**Keywords:** Atrial fibrillation, catheter ablation

## Abstract

Careful patient selection and optimization of the management of active medical conditions prior to proceeding with catheter ablation for atrial fibrillation (AF) is critical to reducing complications and improving ablation success. AF ablation performed on patients who have not been offered appropriate antiarrhythmic drug therapy must be tempered with the procedure risks, particularly for those patients having multiple comorbidities. The inability to comply with systemic anticoagulation for thromboembolic prophylaxis in AF is a contraindication to AF ablation, as premature termination of anticoagulation therapy can lead to catastrophic thromboembolic complications. Successful pulmonary vein isolation (PVI), the cornerstone of AF ablation, is demonstrated by entrance and exit block post ablation, with sustained absence of atrium-to-pulmonary vein conduction in both directions. Beyond PVI, there is no consensus for other endpoints for AF ablation, particularly in patients with persistent or longstanding persistent AF. Complications of PVI for AF have decreased in recent years as technology and knowledge in this field has evolved; however, the risks of cardiac tamponade, thromboembolic complications, esophageal injury, and pulmonary vein stenosis may still be formidable.

## Selecting and preparing the patient for ablation

A critical aspect of success with atrial fibrillation (AF) ablation is careful patient selection and appropriate patient education prior to the procedure. The vexing reality that current therapies, neither medical nor invasive, offer no cure for AF is one that must be reinforced to patients. How we define “ablation success” also has to be made clear, with the ideal goal being arrhythmia attenuation and symptom relief, rather than arrhythmia eradication. Similarly, AF ablation performed on patients who have not been offered appropriate antiarrhythmic drug therapy in an equitable way, prior to AF ablation consideration, must be tempered with the procedure risks, particularly among patients with multiple comorbidities.

### Critical medical problems

Given the infinitesimal possibility of an urgent need to proceed with AF ablation, it is imperative to optimize active medical conditions well before proceeding with catheter ablation. Conditions such as decompensated heart failure, unstable angina, or critical aortic stenosis must be stabilized prior to AF ablation consideration. Likewise, active bronchospasm from emphysema and/or bronchial asthma also needs to be alleviated prior to AF ablation scheduling.

### Obesity

Obesity is a known independent risk factor for AF,^1^ and it is not uncommon for obesity and AF to coexist, given the epic prevalence of the former today. Though the exact mechanism through which obesity contributes to AF has not been clarified, studies such as the LEGACY trial^2^ clearly demonstrated that in overweight or obese AF patients, sustained weight loss is associated with a significant reduction of AF burden and a higher prevalence of sinus rhythm maintenance. The Framingham Heart and Framingham Offspring studies showed that obesity was associated with a 50% increase in the risk of AF, with obesity being independently predictive of AF recurrence.^3^ However, the efficacy of AF ablation among obese patients is yet to be clarified.^4^ Patients with increased body mass index who required prolonged time for the completion of pulmonary vein isolation (PVI) were at greater risk for the development of complications because of their comorbid conditions.^5^ Additionally, mechanical issues leading to high complications, such as difficulty with endotracheal intubation, possible hemodynamic intolerance to general anesthesia, vascular access issues, and substantially higher radiation exposure, remain significant concerns to be addressed.

### Antiplatlet therapy

A substantial proportion of patients undergoing AF ablation have concomitant drug-eluting coronary stents and use dual antiplatelet therapy. Though the risk of bleeding is small, the management of cardiac tamponade or pericardial effusion because of perforation when the patient is on both aspirin and clopidogrel, intuitively, may be more difficult to manage. Our practice is to defer AF ablation until the patient has completed the requirement for dual antiplatelet consumption. This is congruent with the European Heart Rhythm Association/European Society of Cardiology guidelines that recommend that AF ablation should not be performed in patients on aspirin and clopidogrel because of an increased risk of major bleeding secondary to cardiac tamponade, and that AF ablation should be postponed to a time at which aspirin and clopidogrel can be safely discontinued.^6^

### Anticoagulation therapy

The inability to comply with systemic anticoagulation for thromboembolic prophylaxis is a contraindication to AF ablation, as premature termination of anticoagulation therapy can lead to catastrophic thromboembolic complications. Additionally, guideline recommendations now stipulate performing AF ablation with uninterrupted anticoagulation, as this minimizes the risk of periprocedural thromboembolic events. This recommendation was in part put forth through studies such as the COMPARE trial,^7^ Re-Circuit study,^8^ and Venture-AF.^9^ The COMPARE trial investigators showed that AF ablation without warfarin discontinuation reduces the occurrence of periprocedural stroke and minor bleeding complications compared with “bridging” with low-molecular-weight heparin. The Re-Circuit trial was a head-to-head comparison study on the performance of AF ablation on patients receiving uninterrupted dabigatran versus uninterrupted warfarin that found that the incidence of major bleeding events during and up to eight weeks following ablation was significantly lower among the study patients taking dabigatran than those taking warfarin. Uninterrupted anticoagulation using rivaroxaban compared with that using warfarin was evaluated in the Venture-AF study, which reported the occurrence of one bleeding event, one ischemic stroke, and one vascular death, respectively, all of which occurred in the warfarin arm. Consequently, a strategy of performing AF ablation on patients receiving uninterrupted anticoagulation is recommended, and further minimizes the risk of thromboembolic events. Although further study data are needed to best define the efficacy and safety of performing catheter ablation on patients taking uninterrupted Factor Xa inhibitors or direct thrombin inhibitors, there appears to be robust multicenter data in existence to impart a class I recommendation ablation with uninterrupted dabigatran (Class 1, level of evidence (LOE) A) or rivaroxaban (Class 1, LOE B), and a 2A recommendation for the other Xa inhibitors, for which specific clinical studies have either not been performed or are currently underway at this time.^10^

Regarding anticoagulation during catheter ablation, heparin should be administered prior to or immediately following transseptal puncture during AF catheter ablation procedures, and should be adjusted to achieve and maintain an activated clotting time (ACT) of at least 300 s (Class I, LOE B).^10^ A heparin loading dose should be administered initially, followed by a standard heparin infusion. This year’s consensus guidelines^10^ on Catheter and Surgical Ablation of Atrial Fibrillation recommend that the ACT level be checked at 10- to 15-min intervals until therapeutic anticoagulation is achieved, and then at 15- to 30-min intervals for the duration of the procedure. In general, patients receiving a vitamin K antagonist often require less heparin and reach the target ACT faster than those patients taking newer oral anticoagulants, which may necessitate more frequent ACT monitoring and higher heparin doses to be used.^11^

The duration of anticoagulation therapy after an apparent successful AF ablation remains debatable. It is well known that AF ablation appears to convert symptomatic AF to asymptomatic AF. It is not uncommon to see patients who feel great after ablation but remain in persistent AF. Another enigma is that the minimal duration of AF that leads to intracardiac thrombus formation remains unknown. For example, it is not clear if anticoagulation should be continued if a patient experiences occasional AF recurrences lasting three hours each time.

### Antiarrhythmic therapy

Prior to the ablation of supraventricular or ventricular tachyarrhythmias, antiarrhythmic drugs (AADs) are typically discontinued for four to five doses prior to the procedure, in order to enhance arrhythmia inducibility. A similar practice has been adopted for AF ablation in the hope that triggers and/or reentrant atrial tachycardia can be induced during AF ablation procedures. Some existing data show that, in patients with persistent AF and prior AAD therapy failure, the initiation of dofetilide therapy (median: 85 days) prior to AF ablation led to better ablation outcomes and a shortening of the P-wave duration, indicating that favorable remodeling caused by dofetilide may underlie the improved success of AF ablation in these patients.^12^ Recently, the Pulmonary Vein Isolation with versus without Continued Antiarrhythmic Drug Treatment in Subjects with Recurrent Atrial Fibrillation (POWDER-AF) trial^13^ demonstrated that for patients who were free from arrhythmia at three months after PVI, and who continued the use of AADs, also showed lower rates of repeat ablation (hazard ratio (HR): 0.053; p = 0.004) and unscheduled visits (HR 0.055; p = 0.005) without a significant reduction in quality of life. These data indicate that the continuation of AAD therapy post PVI up to 12 months increased the proportion of patients free of atrial tachyarrhythmia from 78.1% off-AAD to 97.3% on-ADD (p < 0.001).

## Procedure-related issues

As with most elective procedures, catheter ablation complications may potentially obviate any would-be benefits of the procedure. Improved procedure-related knowledge, such as the avoidance of ablation within the pulmonary veins (PVs), the use of three-dimensional (3D) electroanatomic mapping with ultrasound, and the maintenance of uninterrupted anticoagulation have all helped to improve complication rates or to at least mitigate their extent, should they occur. Generally, acute complications may be related to vascular access; transseptal puncture; and catheter manipulation, particularly within the left atrium, and particularly during the ablation application itself. Sequelae of these actions may manifest as a vascular pseudoaneurysm, pericardial effusion/tamponade, or embolic phenomena that may lead to neurologic complications, respectively. Similarly, late complications typically include the development of persistent post-PVI atrial tachycardias. Our practice typically employs the use of a micropuncture needle/kit (Cook Medical, Bloomington, IN, USA) and/or vascular ultrasound to achieve vascular access prior to upsizing the system for standard size sheaths. Judicious use of fluoroscopy with the movement of catheters in critical areas or of stiff/bulky catheters such as intracardiac echocardiography (ICE) catheters may decrease the likelihood of additional vascular damage. ICE catheters for access into the left atrium as well as for gathering supplemental data from ultrasound-guided 3D electroanatomic mapping for ablation delivery are widely used. Finally, if linear ablation is used to target roof-dependent or mitral annular atrial tachycardias, then bidirectional block is a prerequisite endpoint to minimize to reduce the risk of development of post-ablation atrial tachycardias.

### Transseptal puncture

The transseptal puncture site should be accurately targeted to the posteriorinferior region of the interatrial septum, an occurrence which is important not only to reduce the risk of complications but also to facilitate catheter delivery to the inferior aspect of the left atrium and PV antrum, particularly the right inferior PV. We commonly utilize standard electrophysiology diagnostic catheters in the coronary sinus and in the His-bundle position, which facilitates approximating the left atrial lateral wall and the most inferior aspect of the non-coronary aortic cusp, respectively. Although the left anterior oblique projection is often used to guide the final push of the transseptal needle across the interatrial septum, it is the right anterior angle projection that helps to differentiate whether the needle is pointing anteriorly towards the aortic root, or posteriorly towards the posterior wall of the right atrium.

Imaging with ICE helps facilitate the diagnosis of the aforementioned problems. The first step in a successful transseptal puncture is engagement of the fossa ovalis. Notably, the inability to do this is not unusual in patients with a markedly dilated right atrium. Utilizing a Brockenbrough needle with an accentuated curve or which demonstrates extra bending may be necessary for successful fossa ovalis engagement in these patients. The next potential hurdle is the inability of the needle to penetrate the fossa ovalis. Successful transseptal puncture can be a challenge in patients who have undergone repeated transseptal catheterization. In such cases, or sometimes de novo, there may be a fibrous or thicker than normal interatrial septum posing undue resistance to puncture. Other septal pathology such as an aneurysmal interatrial septum (primum), dilated atria, or lipomatous hypertrophy may also impart challenges when utilizing the Brockenbrough method to access the left atrium. All of these potential anatomic issues may cause difficulty with advancing the dilator over the needle into the left atrium and, likewise, difficulty in advancing the sheath over the dilator into the left atrium.

It is not uncommon for some operators to exert increased mechanical force to enable the transseptal apparatus (needle/dilator/sheath) to cross the septum; however, the apparatus may surge forward once finally across the septum and may perforate the left atrial free wall. Likewise, the transseptal needle may also slide off of the septum and perforate the right atrium when such forceful pressure is applied. Close analysis performed with ICE imaging and the use of sharp-tip needles (BRK XS or BRK-1 XS; Abbott Laboratories, Chicago, IL, USA); SafeSept^®^ nitinol guidewire (Pressure Products Medical Supplies, Inc., San Pedro, CA); or an NRG^®^ transseptal needle (Baylis Medical, Montreal, Quebec, Canada), which delivers radiofrequency (RF) energy through a closed-tip needle-like device that can help to facilitate left atrial access.

Sometimes, the transseptal sheath is difficult to cross the fossa ovalis when the guidewire is positioned in the left superior or inferior PV. In this case, the force of pushing the transseptal dilator and sheath is built up at the right side of the septum, leading to “buckling” of the sheath/dilator. In this situation, our practice is to position the guidewire or catheter in the right superior PV. In this way, the force is directed upward, and can be transmitted easily along the axis of the sheath/dilator.

### Circumferential PVI

Successful PVI, the cornerstone of AF ablation, is demonstrated by entrance and exit block post ablation, with sustained absence of atrium-to-PV conduction in both directions. Entrance block is evidenced by the absence of PV potential within the isolation line or by the presence of completely dissociated PV potential during atrial pacing or sinus rhythm.^14–16^ Exit block is evidenced by the absence of atrial capture during high-output pacing delivered within the circumferential ablation line.^17,18^ Beyond PVI, there is no consensus for other endpoints for AF ablation, particularly in patients with persistent or longstanding persistent AF. Although, other additional supplementary approaches have been proposed to increase the rate of long-lasting PVI. For example, the incorporation of at least a 20-min waiting period following initial vein post-RF ablation^10^ (Class IIa, LOE B). Similarly, consensus guidelines recommend that adenosine infusion “may be considered” after the waiting period post-initial PVI with radiofrequency ablation^10^ (Class IIa, LOE B). This recommendation centers upon the observation that the administration of adenosine or adenosine triphosphate (ATP), which hyperpolarizes the resting membrane potential of cardiomyocytes, thereby facilitating conduction, has been utilized after acute PVI to unmask dormant PV atrial conduction.^19^ Such reports indicated that additional ablation procedures guided by adenosine or ATP injection resulted in improved long-term success rates for AF ablation.

Although isoproterenol infusion can be used to identify non-PVI triggers in paroxysmal and persistent AF, its role appears limited and there is no consensus requirement to administer isoproterenol for the appraisal of pulmonary vein reconnection post PVI.^20–23^ For persistent AF, additional non-PV targets have been suggested for ablation. For example, the ablation of complex fractionated atrial electrograms (CFAE), and more recently, the ablation of localized rotational activations (so-called rotational activity) are proposed as additional ablation targets, although many recent randomized studies and meta-analyses have not concluded that there is any benefit.^24,25^ Similarly, the answer to whether ablation “homogenization” (ie, radiofrequency ablation to eliminate all residual electrograms) should be performed for persistent AF remains unclear.^26^

### Ablation lesion formation

Theoretically, successful ablation is contingent on the generation of consistently transmural lesions that block the propagation of rapidly firing PV or non-PV triggers, as well as eliminate the substrate responsible for reentry. The size of RF energy lesions is a function of power, impedance, temperature, duration, and catheter contact force.^27–29^ In an effort to mitigate the RF energy from the posterior left atrial wall to the thin-walled esophagus and because there is no other well-established method to prevent esophageal injury, the consensus guidelines recommend the use of an esophageal temperature probe during PVI ablation to monitor esophageal temperature and help guide energy delivery (Class IIa, LOE C).^10^

As the wall thickness of different regions of the left atrium varies substantially, it is a balancing act to create effective transmural lesions at multiple sites without perforation. Though contact force is an integral component in the successful creation of ablation lesions, there is no consensus that the use of a contact force catheter imparts better efficacy for PVI. Additionally, a recent randomized head-to-head comparison study that sought to see if contact force improved results demonstrated that, despite contact force data being associated with reduced acute PV reconnection, it did not improve one-year success rates.^30^ Though contact force information is helpful, many operators prefer other options because contact force catheters are often stiff and unwieldy. If an operator does choose to use a contact force catheter, then expert guidelines recommend a starting point minimum targeted contact force of 5 g to 10 g (Class IIa, LOE C).^10^

## Data on complications reported in published studies

The major studies that summarize cumulative and specific complication rates for AF ablation using RF energy are tabulated in **Table 1**. Overall, the complication rates are low (most range from 3% to 7%). The pericardial effusion or tamponade complication rate ranged from 1% to 3%. Vascular injury and neurologic complications ranged from less than 1% for both to just over 5% and 6%, respectively. Reported esophageal injuries were less than 0.20%, and the reported death rate was less than 1%. However, when atrioesophageal fistula occurs, the mortality rate can be as high as 50% to 100%. The PV stenosis rate was estimated to be as high as 1.63% in the 1995 to 2002 study period, but decreased to ≤ 0.5% among most studies with time, likely due to the widely adopted ablation technique of large-area circumferential ablation.

Since the introduction and approval of the cryoballoon ablation technology for PVI by the United States and European regulatory agencies, cryoballoon ablation has been widely adopted in use by electrophysiologists. The FIRE AND ICE multicenter randomized controlled trial compared the safety profile as well as the acute and long-term efficacies of the second-generation cryoballoon (Artic Front Advance™, Medtronic, USA) in comparison with conventional RF PVI.^31^ The results indicated the non-inferiority of cryoablation technology compared with RF-based ablation with respect to efficacy and the safety of patients with drug-refractory AF.^32^ In a subgroup analysis of the secondary endpoints, such as rehospitalization, a requirement for cardioversion, and repeat PVI, cryoballoon performance appeared to be a slightly better option.^33^ Maan et al.^34^ did an extensive review of AF ablation complications, including of cryoballoon-associated complications, and reported that the most frequent complication was phrenic nerve palsy, which is specifically more likely to occur when a smaller 23 mm (rather than 28 mm) balloon is used to isolate the right superior PV.^35–37^ Other critical complications such as esophageal injury have been reported, particularly when cryo applications were delivered to the left inferior PV.^38^ With a lesser number of cryo applications and a shorter duration (<180 s) of each cryo application, the risk of atrioesophageal fistula is expected to be lower in the future; however, there is less clinical experience with this technology than experience with RF ablation.

Three recent, reasonably large studies that specifically address second-generation cryoablation complications are summarized in **Table 2**. The most frequent complications were phrenic nerve palsy or vascular access injury. Chun et al.^39^ reported a statistically lower risk of tamponade among the cryoballoon PVI group than among the RF PVI group. This may in part be related to the requirement for only a single transseptal puncture to be performed, compared with two punctures, for more instances of RF PVI. However, they also demonstrated that ablation beyond PVI was an independent risk factor for cardiac tamponade. The study by Guhl et al.^40^ systematically demonstrated an increased risk for phrenic nerve injury with the use of the 23-mm cryoballoon as well as with advanced age. The injury was transient for the majority of their cohort, and they noted that recommendations of monitoring diaphragmatic electromyographic signals with compound motor action potential^41–45^ and rapid balloon deflation or double deflation leading to a more rapid tissue warming may reduce the extent of phrenic nerve injury.^46^ The study by Mugnai et al.^47^ reported that the overall complication rate using second-generation cryoballoon PVI was 2%, with vascular access being a frequent major complication. Thromboembolic complications were estimated to be 0.2% and, though phrenic nerve palsy was found in 7.2% of patients, it was transient in the vast majority of these individuals like in the other reported studies. The authors attributed vascular injury as possibly secondary to the use of a 15-French FlexCath^®^ sheath (Medtronic, Minneapolis, MN, USA). Despite some of these technical limitations, cryoablation appears to be an effective alternative to RF catheter ablation for AF.

## Conclusions

Complications of PVI for AF have decreased in recent years as technology and knowledge in this field has evolved; however, the risks of cardiac tamponade, thromboembolic events, esophageal injury, and PV stenosis may still remain formidable. PVI is the cornerstone of AF ablation, and the patient must be educated that the realistic goal is arrhythmia attenuation rather than eradication. The main hurdle of better ablation success is the lack of understanding of the multiple mechanisms underlying the initiation, maintenance, and progression of AF. To date, new ablation technologies (contact force catheters, cryoballoons, rotor mapping) have all failed to significantly improve ablation success. Additional ablation lesion sets, such as CFAE and linear lesions, have also failed to improve ablation success. Mitigating procedure-related complications is therefore paramount.

## Figures and Tables

**Table 1:** RF Cathetero Ablation Complication Studies

Study Period	Article	Mean Age	Sample Size/ Number of RF Ablations Analyzed	LA Diameter LVEF Gender Demographics	Study Type and Location	Overall Complication Frequency	Specific Complications Reported
1995–2002	Cappato et al. 2005^48^	N/A	12,830	LA Diameter: < 4.63 cm LVEF: > 64.3% %Female: N/A	Worldwide Survey	5.90%	Death: 0.05% Pericardial complications: 1.22% Cardiac surgery: 0.03 Myocardial infarction: not reported Neurologic complications: 0.98% Vascular injury: 0.95% Pulmonary vein stenosis: 1.63% Hemo/Pneumothorax: 0.18% Esophageal injury: not reported Heart block: not reported Mitral valve injury: 0.01% Phrenic nerve injury: 0.11% Infectious complications: 0.01% Respiratory complications: not reported
2000–2010	Deshmukh et al. 2013^49^	18 to >80 years	93,801	LA Diameter: N/A LVEF: N/A %Female: 40%	Nationwide Inpatient Sample (NIS); USA	6.29%	Death: 0.42% Pericardial complications: 1.52% Cardiac surgery: 0.28% Myocardial infarction: 0.37% Neurologic complications: 1.02% Vascular injury: 1.53% Pulmonary vein stenosis: not reported Hemo/Pneumothorax: 0.39% Esophageal injury: not reported Heart block: not reported Mitral valve injury: not reported Phrenic nerve injury: not reported Infectious complications: 0.38% Respiratory complications: 1.30%
2000–2012	Gupta et al. 2013^50^	57.3 years	83,000	LA Diameter: 4.19 cm LVEF: 58.5% %Female: 25%	MEDLINE/Embase Review; International	2.9%	Death: 0.06% Pericardial complications: 1.70% Cardiac surgery: not reported Myocardial infarction: not reported Neurologic complications: 0.80% Vascular injury: 2.30% Pulmonary vein stenosis-0.50% Hemo/Pneumothorax: 0.40% Esophageal injury: 0.08% Heart block: not reported Mitral valve injury: 0.20% Phrenic nerve injury: 0.40% Infectious complications: 0.10% Respiratory complications: not reported
2001–2007	Spragg et al. 2008^51^	57.2 years	641	LA Diameter: 4.7 cm LVEF: 57% %Female: 22.3%	Single-center; Johns Hopkins Medical Center, Baltimore, MD, USA	5%	Death: 0% Pericardial complications: 1.2% Cardiac surgery: 0.16% Myocardial infarction: 0% Neurologic complications: 1.1% Vascular injury: 1.7% Pulmonary vein stenosis: 0.16% Hemo/Pneumothorax: 0.31% Esophageal injury: 0% Heart block: 0.16% Mitral valve injury: 0.16% Phrenic nerve injury: not reported Infectious complications: 0% Respiratory complications: 0%
2001–2006	Ellis et al. 2009^52^	N/A	6,065	LA Diameter: N/A LVEF: N/A %Female: N/A	Medicare Provider Analysis and Review (MEDPAR) Data Review; USA	9.1%	Death: 0.48% Pericardial complications: 2.82% Cardiac surgery: not reported Myocardial infarction: not reported Neurologic complications: 0.62% Vascular injury: 5.37% Pulmonary vein stenosis: not reported Hemo/Pneumothorax: 0.33% Esophageal injury: not reported Heart block: not reported Mitral valve injury: not reported Phrenic nerve injury: not reported Infectious complications: not reported Respiratory complications: not reported
2001–2010	Hoyt et al. 2011^53^	58.5 years	1,190	LA Diameter: 4.6 cm LVEF: 57% %Female: 24.6%	Single-center; Johns Hopkins Medical Center, Baltimore, MD, USA	4.7%	Death: 0% Pericardial complications: 1.10% Cardiac surgery: 0% Myocardial infarction: 0% Neurologic complications: 1.10% Vascular injury: 1.50% Pulmonary vein stenosis: 0.1% Hemo/Pneumothorax: 0.2% Esophageal injury: 0% Heart block: 0.1% Mitral valve injury: 0.1% Phrenic nerve injury: 0.3% Infectious complications: not reported Respiratory complications: not reported
2003–2006	Cappato et al. 2010^54^	N/A	16,309	LA Diameter: 3.18 cm LVEF: > 22.4% %Female: 39.2%	Worldwide Survey	4.54%	Death: 0.15% Pericardial complications: 1.31% Cardiac surgery: not reported Myocardial infarction: not reported Neurologic complications: 0.94% Vascular injury: 1.47% Pulmonary vein stenosis: 0.29% Hemo/Pneumothorax: 0.11% Esophageal injury: 0.04% Heart block: not reported Mitral valve injury: 0.07% Phrenic nerve injury: 0.17% Infectious complications: 0.01% Respiratory complications: not reported
2003–2010	Winkle et al. 2012^55^	61.4 years	1,503	LA Diameter: 4.3 cm LVEF: N/A %Female: 28.5%	Single-center; Sequoia Hospital, Redwood City, CA, USA	3.00%	Death: not reported Pericardial complications: 0.70% Cardiac surgery: not reported Myocardial infarction: not reported Neurologic complications: 0.30% Vascular injury: 0.90% Pulmonary vein stenosis: 0.07% Hemo/Pneumothorax: not reported Esophageal injury: 0.07% Heart block: 0.07% Mitral valve injury: not reported Phrenic nerve injury: not reported Infectious complications: 0.20% Respiratory complications: 0.13% Total complications: 3.00%
2005–1008	Shah et al. 2012^56^	61.7 years	4,156	LA Diameter: N/A LVEF: N/A %Female: 29.3%	Multicenter, California State Inpatient Database Review; CA, USA	5.1%	Death: 0.50% Pericardial complications: not reported Cardiac surgery: not reported Myocardial infarction: not reported Neurologic complications: 6.10% Vascular Injury: not reported Pulmonary vein stenosis: not reported Hemo/Pneumothorax: 1.90% Esophageal injury: not reported Heart block: not reported Mitral valve injury: not reported Phrenic nerve injury: not reported Infectious complications: not reported Respiratory complications: not reported
2007–2009	Piccini et al. 2012^58^	72 years	15,423	LA Diameter: N/A LVEF: N/A %Female: 41%	Medicare Beneficiaries Database Review; USA	4.4%	Death: 0.80% Pericardial complications: 1.90% Cardiac surgery: not reported Myocardial infarction: 0.30% Neurologic Complications: 0.90% Vascular injury: 0.50% Pulmonary vein stenosis: not reported Hemo/Pneumothorax: not reported Esophageal injury: not reported Heart block: not reported Mitral valve injury: not reported Phrenic nerve injury: not reported Infectious complications: not reported Respiratory complications: not reported
2010–2015	Chun et al. 2017^39^	67 years	2,125	LA Diameter: 4.2 cm LVEF: 58% %Female: 39%	Single-center; Cardioangiologisches Centrum Bethanien, Frankfurt, Germany	2.9%	Death: 0% Pericardial complications: 1.50% Cardiac surgery: not reported Myocardial infarction: not reported Neurologic complications: 0.20% Vascular injury: 2.60% Pulmonary vein stenosis: 0% Hemo/Pneumothorax: 0.09% Esophageal injury: 0.05% Heart block: not reported Mitral valve injury: not reported Phrenic nerve injury: 0% Infectious complications: not reported Respiratory complications: not reported

N/A: not applicable, not reported, or unable to determine; LA: left atrial/atrium; LVEF: left ventricular ejection fraction; RF: radiofrequency.

**Table 2:** Second-generation Cryoballoon Complication Studies

Study Period	Article	Mean Age	Sample Size/ Number of RF Ablations Analyzed	LA Diameter LVEF Gender Demographics	Study Type and Location	Overall Complication Frequency	Specific Complications Reported
2010–2015	Chun et al. 2017^39^	64 years	875	LA Diameter: 4.0 cm LVEF: 61% %Female: 41%	Single-center; Cardioangiologisches Centrum Bethanien, Frankfurt, Germany	5.4%	Death: 0% Pericardial complications: 0.1% Cardiac surgery: not reported Myocardial infarction: not reported Neurologic complications: 0.4% Vascular injury: 2.9% Pulmonary vein stenosis: 0% Hemo/Pneumothorax: 0.2% Esophageal injury: 0% Heart block: not reported Mitral valve injury: not reported Phrenic nerve injury: 1.7% Infectious complications: not reported Respiratory complications: not reported
2011–2015	Guhl et al. 2016^40^	59 years	450	LA Diameter: 4.0 cm LVEF: 55% %Female: 26%	Single-center; University of Pittsburgh Medical Center, Pittsburgh, PA, USA	2.2%	Death: 0% Pericardial complications: 0.67% Cardiac surgery: not reported Myocardial infarction: not reported Neurologic complications: not reported Vascular injury: 0.22% Pulmonary vein stenosis: not reported Hemo/Pneumothorax: not reported Esophageal injury: not reported Heart block: not reported Mitral valve injury: not reported Phrenic nerve injury: 10.8% Infectious complications: not reported Respiratory complications: not reported
2012–2015	Mugnai et al. 2015^47^	58 years	500	LA Diameter: 4.2 cm LVEF: 59% %Female: 33%	Single-center; Heart Rhythm Center, UZ, Brussels, Belgium	2.0%	Death: 0% Pericardial complications: 0.2% Cardiac surgery: not reported Myocardial infarction: not reported Neurologic complications-: 0.2% Vascular injury: 1.4% Pulmonary vein stenosis: 0% Hemo/Pneumothorax: not reported Esophageal injury: not reported Heart block: not reported Mitral valve injury: not reported Phrenic nerve injury: 7.2% Infectious complications: not reported Respiratory complications: not reported

N/A: not applicable, not reported, or unable to determine; LA: left atrial/atrium; LVEF: left ventricular ejection fraction; RF: radiofrequency.
